# Species Diversity within a Community of the Curcurbit Fruit Flies *Bactrocera cucurbitae, Dacus ciliatus,* and *Dacus demmerezi* Roosting in Corn Borders Near Cucurbit Production Areas of Reunion Island

**DOI:** 10.1673/031.012.3201

**Published:** 2012-03-01

**Authors:** J.-P. Deguine, T. Atiama-Nurbel, E. Douraguia, F. Chiroleu, S. Quilici

**Affiliations:** CIRAD, UMR PVBMT ClRAD/University of La Réunion, 7 chemin de l'Irat, Ligne Paradis, 97410 Saint-Pierre (La Réunion), France

**Keywords:** relative abundance, seasonal abundance, sex ratio, Tephritidae

## Abstract

In order to better control fruit flies (Diptera: Tephritidae) attacking Cucurbitaceae on Reunion Island (21°6 S/ 55°36 E), biological characteristics (seasonal fluctuation, relative abundance, sex ratio) of communities roosting in corn borders were investigated. The study was conducted in austral summer across a range of altitudes (750–1150 m) corresponding to the main areas of cucurbit cropping. Living adults were recorded roosting on corn planted within or around cucurbit fields. Results showed a high variability in seasonal fluctuation of populations according to local conditions. *Bactrocera Cucurbitae* (Coquillett) (Diptera: Tephritidae) was the least abundant species (27%) compared to *Dacus ciliatus* Loew (36%) and *Dacus demmerezi* Bezzi (37%). Relative abundance of *B. Cucurbitae* was lowest (< 18%) in high altitude sites (above 1000 m), where *D. demmerezi* was the most prevalent species (> 56%). *Dacus ciliatus* showed variable relative abundance (from 18 to 51%) depending on the experimental design (varying in location and in year). Sex ratio was also very variable from one species to another and from one experimental design to another.

## Introduction

As Tephritidae is the most damaging family of Diptera for agriculture worldwide, many studies have been devoted to their bioecology. Reviews, such as the one by White and Elson-Harris (1992), are numerous and provide a basic background to propose integrated pest management methods for these pests. However, few studies deal with the characteristics of communities including several species, especially those attacking Cucurbitaceae (Dhillon et al. 2005). In addition, most of the knowledge is based on laboratory studies or field experiments using either male parapheromone or food-baited traps or collections of infested fruit.

On Reunion Island (21°6′ S/ 55°36′ E), various studies have been conducted on Tephritid species attacking fruit (Quilici 1994; Duyck et al. 2008; Rousse et al. 2006) or tomato (Brévault and Quilici 2007, 2009), but fewer studies have focused on the three species attacking Cucurbitaceae: *Bactrocera Cucurbitae* (Coquillett) (Diptera: Tephritidae), *Dacus ciliatus* Loew, and *Dacus demmerezi* Bezzi (Vayssières 1999; Vayssières and Carel 1999; Vayssières et al. 2008). Most of these studies were devoted to fly biology in the laboratory or dealt with distribution or damage of larvae by collecting infested fruit or adult dynamics using sexual traps. Little is known of the bioecology of the populations and communities of adults living in the fields.

**Figure 1.**
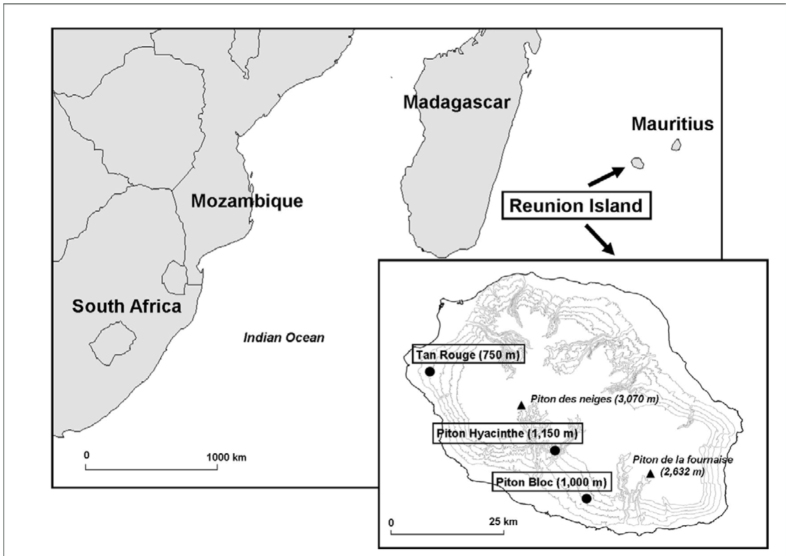
Location of Reunion Island in the Indian Ocean and locations of the three experimental sites on the island with their altitude. Research years per site: Piton Bloc (2008), Piton Hyacinthe (2009), and Tan Rouge (2009 and 2010). High quality figures are available online.

Adults of *B. cucurbitae* are known to spend much time on roosting sites such as corn (Nishida and Bess 1957; McQuate and Vargas 2007). In order to study the characteristics of the communities of these three species in the field, we recorded the number of adult flies roosting on corn planted within or around fields of cultivated cucurbits. Our specific objectives were to study in different experimental designs the seasonal fluctuation of the communities, the relative abundance of the species, and their sex ratio.

To our knowledge, no other study has focused on populations of *B. cucurbitae*, *D. ciliatus*, and *D. demmerezi* together using field records of adult populations. This study should contribute to the implementation of sustainable protection methods against cucurbit fruit flies.

## Materials and Methods

### Description of sites

The study was conducted during austral summer from 2008 to 2010 at three sites (Figure 1): Piton Bloc (21°18′ S, 55°34′ E), at an altitude of 1000 m above sea level; Piton Hyacinthe (21°13′ S, 55°31′ E), at 1150 m; and Tan Rouge (21°03′ S, 55° 18′ E) at 750 m. Four experimental designs (varying in location and/or year) were considered during these three years, with the latter site being considered both in 2009 and 2010. The sites were considered across a range of altitudes (750 m to 1150 m) corresponding to major cucurbit crop areas. In the following text, the abbreviation PB08 will be used for Piton Bloc (2008), PH09 for Piton Hyacinthe (2009), TR09 for Tan Rouge (2009), and TR10 for Tan Rouge (2010). These were chosen for their similarity in agroecosystem characteristics (field where cucurbit was grown; plantation of corn within or around the field acting as a roosting site or trap plant), climatic conditions (average temperature, average humidity), and timing of crop plantation and growth during austral summer. However, some differences have to be mentioned: zucchini (*Cucurbita pepo*) was grown in three experimental designs and pumpkin (*Cucurbita moschata*) in the fourth one (TR09); the size of the fields differed from one situation to another one; and the design of trap corn varied (border, strip, and/or patch). Table 1 gives cultural information both for cucurbit and corn plantation. All fields were untreated.

### Observations of adult flies

The study focused on wild adults of the three Cucurbit fruit fly species present on Reunion Island: *B. Cucurbitae, D. ciliatus,* and *D. demmerezi*. The observations were carried out several times per experimental design, ranging from three in PB08 to eight in TR10. The observations consisted of recording wild flies roosting on corn plants and distinguishing species and sex for each adult observed. During observations, flies were carefully approached and were not disturbed while being observed. Each observation was done with the exact same protocol in the different experimental designs although the size of observed corn plants and time allocated to observations were different according to the design of the cucurbit field and the shape of corn plantation. Sections of corn (sample units for observations) were delimited as follows: 20 sections of 1 m^2^ in PB08; 33 sections of 2 m^2^ in PH09; nine sections of 1 m^2^ in TR09; and 54 sections of 1 m^2^ in TR10. Flies were counted on all corn plants located in a section. In the experiments, the time allocated to one hourly observation of flies roosting on corn varied from 12 min (TR09) to 50 min (PH09) according to the area of corn around or within the field (Table 1). Three people carried out each observation: two observers provided fly numbers and one person recorded numbers provided by the observers. A total of 147 hours were devoted to fly records during this study.

### Variables considered

Three community characteristics were considered in this study: the number of adult flies, the relative abundance, and the sex ratio. In order to compare the characteristics of the communities in the different experimental designs, we chose a criterion that could take into account two major possible biases. First, it had to take into account the daily patterns of the fly species and the daily variation in population level from one hour to another. Thus, the total number of flies during the 12 daily observations (distinguishing species and sex) was considered. Second, it had to take into account the different sizes of the observed corn areas. To do this, the total number of flies was finally transformed into a daily number of flies per 10 m^2^ of corn; 10 m^2^ corresponded to the smallest common observed corn area (Table 2). This criterion was called density of adults (per 10 m^2^). Otherwise, the relative abundance of a species corresponds to the ratio between the numbers of adults of this species recorded and the number of flies belonging to the three species. For each species, the sex ratio was evaluated by the ratio between the number of females and the number of adults (females and males).

**Figure 2.**
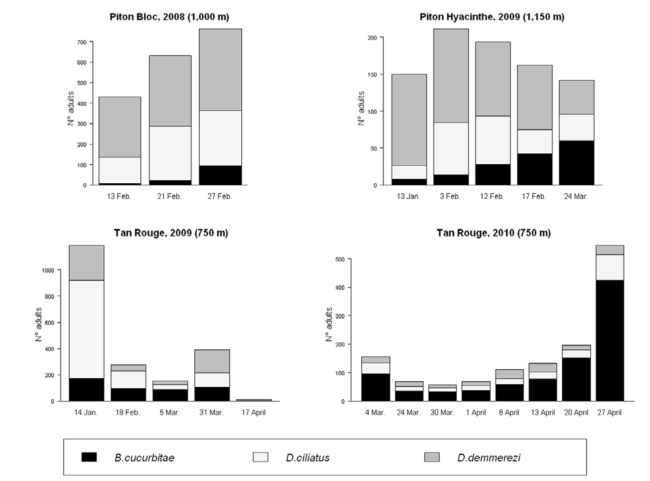
Seasonal fluctuation of the structure of the adult fly community (density per 10 m^2^ of corn per date) in each experimental design. High quality figures are available online.

### Data and statistical analysis

All the graphs and the statistical tests (all at 5% threshold) were done with R version 2.11.1 (R Development Core Team 2010). The tests aimed to display the characteristics of the fly communities. Three types of statistical analysis were used in the study. First, relative abundances of the three species were compared with a pairwise proportion test based on a Chi-squared test with Benjamini and Hochberg correction (Benjamini and Hochberg 1995), both per experimental design and in all of the experimental designs. Second, per year and per site, effects of species on sex ratio were tested through a generalized linear model on binomial data and with a likelihoodratio test. A generalized linear hypothesis test, based on Tukey's HSD test (Hothorn et al. 2008) was performed to compare the average sex ratios between species. Third, an exact binomial test was used in order to determine if the proportion of females was equal to 50%, taking into account all experimental designs and all dates for each species.

## Results

### Period of observation: austral summer

The numbers of observation dates were different from one experimental design to another one, according to the dates of sowing (corn and cucurbit), the period of fruiting of cucurbit, the availability and the vegetative stage of corn in the season. Thus, the period of observations globally covered the cucurbit cropping period (January to April), which also corresponds to the warmest season in the year (austral summer) (Table 2).

### Numbers of flies observed and size of populations

A total of 18,441 adult flies were recorded on corn plants in this study, and the average density of adults (per 10 m^2^) was comparable in the four situations. Table 2 shows the numbers of flies counted per year and per site: from 1819 adults in TR09 to 7227 adults in TR10 (ratio 1:4). The average density of adults per 10 m^2^ per date varied from 167.3 flies in TR10 to 606.7 flies in PB08 (ratio 1:3.6) (Table 2), while the total number of flies per 10 m^2^ (depending of the number of observations per experimental design) varied from 857.91 in PH09 to 2021.12 in TR09 (ratio 1:2.4) (Table 3).

### Seasonal fluctuation of adult populations

The seasonal fluctuation of fly density was variable from one situation to another (Figure 2). In PH09, similar population levels were observed from January to March with a moderate peak in February (about 200 flies per 10 m^2^). In TR09, the number of flies strongly decreased from January (1200 per 10 m^2^) to February (250 per 10 m^2^), and then stayed below 400 flies until April. The situation of TR10 differed from TR09 and the density increased from March (50 to 100 flies per 10 m^2^) to a high peak at the end of April (500 flies per 10 m^2^). In PB08, the period of observation was too short to present a meaningful trend of density evolution.

**Figure 3.**
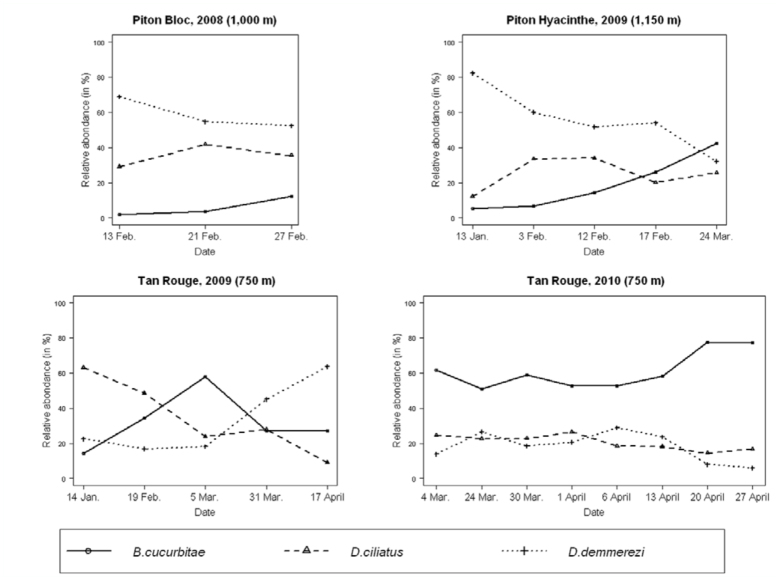
Relative abundances of adults of *Bactrocera cucurbitae, Dacus ciliatus,* and *Dacus demmerezi* in each experimental design. High quality figures are available online.

### Relative abundance of the three species

First, relative abundances of the three species differed considerably from one experimental design to another, although all three species were present in each of the four situations. In each situation, one species represented more than 50% of the total number of flies (Figure 3): *B. cucurbitae* in TR10, *D. ciliatus* in TR09, and *D. demmerezi* in PB08 and in PH09. Figure 2, which also illustrates the change in abundance of these species during the observation period, stresses that the main species in a situation had a significant impact on the change in global number of flies.

The relative abundances of the three species were highly significantly different in each experimental design. Table 4 shows the results of the comparisons of the relative abundances of these species per year and per site. In PB08 (1000 m), *D. demmerezi* (57.05%) was significantly more abundant than *D. ciliatus* (36.20%) (*p* < 0.01), which in turn was significantly more abundant than *B. cucurbitae* (6.75%) (*p* < 0.01). In PH09 (1150 m), the second highest site, *D. demmerezi* (56.30%) was significantly more abundant than *D. ciliatus* (26.05%) (*p* < 0.01), which was significantly more abundant than *B. cucurbitae* (17.65%) (*p* < 0.01). In TR09, *D. ciliatus* was significantly more abundant (50.91%) than *D. demmerezi* (26.06%) (*p* < 0.01), which was significantly more abundant than *B. cucurbitae* (23.03%) (*p* < 0.05). In contrast, in TR10 (same site, following year), *B. cucurbitae* was significantly more abundant (68.20%) than *D. ciliatus* (18.63 %) (*p* < 0.01), which was significantly more abundant than *D. demmerezi* (13.17%) (*p* < 0.01). The relative abundances of each species showed a high variability in the four experimental designs: from 6.75% (PB08) to 68.20% (TR10) for *B. cucurbitae* (difference of 61.45 points), from 18.63% (TR10) to 50.91% (TR09) for *D. ciliatus* (difference of 32.28 points), and from 13.17% (TR10) to 57.05% (PB08) for *D.* *demmerezi* (difference of 43.88 points).

Globally, *D. demmerezi* and *D. ciliatus* were overall the two equally dominant species while *B. cucurbitae* was the less represented one. The overall relative abundances of *D. demmerezi* (36.85%) *and D. ciliatus* (35.79%), but not significantly different (*p* = 0.23), were significantly greater than the overall relative abundance of *B. cucurbitae* (27.36 %) (*p* < 0.01).

### Sex ratio

**Figure 4.**
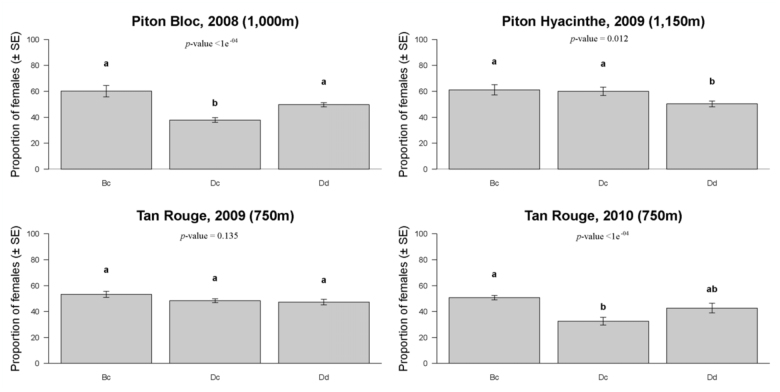
Sex ratio of *Bactrocera cucurbitae* (Bc), *Dacus ciliatus* (Dc), and *Dacus demmerezi* (Dd) in each experimental design. Tests with General Linear Model (with *p* < 0.05) were done separately on adult fly density (per 10 m^2^ of corn), which are given (date was considered as a replication). Standard errors and *p*-values are given for each experimental design. Sex ratios not followed by the same letter in range are significantly different. High quality figures are available online.

First, a considerable variability in the sex ratio (% of females) both between fly species and between experimental designs was observed (Figure 4). In PB08, the effect of species on the sex ratio was highly significant (*p* < 0.01). The sex ratio of *B. cucurbitae* (60.57%) was not significantly different from the sex ratio of *D. demmerezi* (49.66%) (*p* = 0.0679). The sex ratios of these two species were significantly greater than the sex ratio of *D. ciliatus* (37.72%, p < 0.01). In PH09, the effect of species on the sex ratio was significant (*p* < 0.05). The sex ratio of *B. cucurbitae* was not significantly different from *D. ciliatus* (*p* = 0.9797). The sex ratios of *B. cucurbitae* (62.21%) and *D. ciliatus* (60.69%) were significantly greater than the sex ratio of *D. demmerezi* (50.47%) (both *p* < 0.05). In TR09, the effect of species on the sex ratio was not significant (*p* = 0.135), with the following sex ratios: *B. cucurbitae* (53.22%), *D. ciliatus* (48.38%), and *D. demmerezi* (47.46%). In TR10, the effect of species on the sex ratio was very highly significant (*p* < 0.01). The sex ratio of *B. cucurbitae* (50.81%) was significantly greater than the sex ratio of *D. ciliatus* (32.69%) (*p* < 0.01) but not significantly different from the sex ratio of *D. demmerezi* (42.86%) (*p* = 0.1182). The sex ratio of *D. demmerezi* was not different from the sex ratio of *D. ciliatus* (*p* = 0.0834).

On the whole, sex ratios of the three species were compared for all experimental designs. The effect of species on the sex ratio was very highly significant (*p* < 0.01). The sex ratio of *B. cucurbitae* (53.25%) was significantly greater than the sex ratio of *D. demmerezi* (48.78%) (*p* < 0.05) and significantly greater than the sex ratio of *D. ciliatus* (44.59%) (*p* < 0.01). The sex ratio of *D. demmerezi* was significantly greater than the sex ratio of *D. ciliatus* (*p* < 0.05).

Proportion of females was compared to 50% in an exact binomial test, taking into account all experimental designs and all dates for each species. All the results were very highly significant. The sex ratio of *B. cucurbitae* was significantly greater than 50% (*p* < 0.01), the sex ratio of *D. demmerezi* was equal to 50% (*p* = 0.113), and the sex ratio of *D. ciliatus* was significantly less than 50% (*p* < 0.01).

## Discussion

The high density of adults recorded in the four situations (from 167.3 flies in TR 2010 to 607.7 flies in PB 2008 per date and per 10 m^2^ ) confirms that the populations of cucurbit flies are high during austral summer when cucurbit is grown. These observations are in line with the results of other studies conducted in La Réunion, not only on the species of Dacini attacking cucurbits (Vayssières and Carel 1999), but also on Tephritid species attacking fruit (Duyck et al. 2004) and on the Tomato Fly (Brévault and Quilici 2007).

Our study confirms the presence of *B. cucurbitae, D. ciliatus,* and *D. demmerezi* at altitudes between 750 m and 1150 m. This observation is in accordance with data from the literature concerning the altitudinal distribution on *B. cucurbitae* in different parts of the world (Dhillon et al. 2005) and also with the results of Etienne (1972) and Vayssières and Carel (1999) on the three species on Reunion Island. Etienne (1972) specifies that *B. cucurbitae* nearly disappears above 800 m and that *D. demmerezi* is the dominant species between 600 m and 1200 m. Vayssières and Carel (1999), using fruit fly trapping and fruit collections, state that during austral summer *B. cucurbitae* occurs from 0 m to 1200 m, *D. ciliatus* from 0 m to 1400 m and *D. demmerezi* from 500 m to 1600 m. Besides, Vayssières et al. (2008) show that at sea level, 90% of infested fruit collected from wild cucurbit species are infested by *B. cucurbitae,* with *D. ciliatus* and *D. demmerezi* accounting for the remaining 10%, and *B. cucurbitae* is the most abundant species in cultivated plants. In this latter altitude, the competitive advantage of *B. cucurbitae* could be explained by favorable temperature conditions, with shorter egg incubation and larval development periods. This advantage might be less important in our highland zone study (750– 1150 m), which could explain the overall lower proportion of *B. cucurbitae* (27.36%) observed in our study. In addition, for the same cultivated host (zucchini), our study showed that proportion of *B. cucurbitae* was highest (68.20%) in the lowest altitude (TR10, 750 m) and lowest in the highest situations (6.75% in PB08 and 17.65% in PH09). By contrast, the proportion of *D. demmerezi,* usually considered on Reunion Island as a “highland fly” was confirmed in our study to be dominant (57.05% and 56.30%) in the highest sites, Piton Bloc (1000 m) and Piton Hyacinthe (1150 m), respectively. In these two sites, *D. ciliatus* also appeared to be more abundant than *B. cucurbitae.* Those observations are also in line with the conclusions of Vayssières (1999) on the altitudinal range of the three species. They also are in agreement with other studies on the relation between seasonal fluctuations of *B. cucurbitae* and temperature (Banerji et al. 2005; Dhillon et al. 2005; Shivayya and Kumar 2008). Moreover, seasonal abundance also depends on the host range of the fly species and their use of the different hosts, as demonstrated by Clarke et al. (2001) on several species of *Bactrocera* in Thailand and Malaysia. It is probably the case for the three species of Dacini on Reunion Island, which are oligophagous on cultivated and wild cucurbits. In addition to temperature, which plays a key role in the altitudinal occurrence of fruit flies, other abiotic factors (humidity and rainfall) or biotic factors (host plants, natural enemies) can also affect their relative abundance (Garcia 2009). Finally, Sivinski et al. (2004) showed that for some species of *Anastrepha spp.* in Mexico, local changes in the environment of an agroecosystem (habitat characteristics and nature of host plant) had more influence on the relative abundance of the fruit fly species than their competition for egg-laying. This conclusion reinforces our observations on the differences of relative abundances of the three species in the four situations. For example, in our study, *D. ciliatus* was dominant in the only one situation where pumpkin was grown. In Tan Rouge (750 m), the proportion of *D. ciliatus* was only 18.63% in 2010 when zucchini was grown, while it reached 50.91% in 2009 when pumpkin was cultivated. Conversely, the relative abundance of *B. cucurbitae* was 68.20% in TR10 when zucchini was grown, although it was only 23.03% in the same field in 2009 (TR09) when pumpkin was grown. These differences from one year to the other in this site may be explained (i) by the possible preference of *D. ciliatus* for pumpkin compared to zucchini (*D. ciliatus* is also called the Pumpkin Fruit fly), even if it was not reported before on Reunion Island, and (ii) by the preference of *B. cucurbitae* for zucchini, which has already been shown (Vayssières 1999). The change of surrounding wild host fruit between 2009 and 2010 could be also the source of the variation in *B.cucurbitae* population, as already shown in Hawaii (Vargas et al. 1990). Finally, in our study the relative abundances of *B. cucurbitae, D. ciliatus,* and *D. demmerezi* seem to represent cross-results of (i) abiotic global factors, such as temperature correlated with altitude (Vayssières and Carel 1999), (ii) abiotic local factors, such as localized rainfall (Mwatawala et al. 2010), and (iii) biotic local factors, such as host plant (i.e., zucchini vs. pumpkin), roosting plants (availability and quality), or natural enemies. This conclusion suggests that the knowledge of local factors is needed to predict the relative abundances of the three species in a framework of pest management.

Overall, increases or decreases in the size of the communities from the highest site (Piton Hyacinthe, 1150 m) to the lowest one (Tan Rouge, 750 m) were not observed. It is difficult to state much of altitudinal trends when only three altitudes were included, and the total fly numbers at the lowest altitude varied quite a bit between the two years. In addition, some seasonal fluctuations of the populations can be emphasized. In PH 09 the peak observed (3 February 2009) can be linked with the date of maximum presence of zucchini fruit. The population then decreased slowly with the reduction of fruit availability and with the senescence of corn plants (unpublished data). In TR09, the drastic fall in flies observed after 14 January 2009 (mainly *D. ciliatus)* can also be linked with the reduction of availability of pumpkin fruit in the field (unpublished data). At the end of April, an outbreak of *B. cucurbitae,* that could correspond to the emergence of a new generation of adults, was observed on corn plants when zucchini fruit was not available. The end of March 2010 was indeed the period of maximum egg—laying by *B. cucurbitae* females in this situation, and the breakout occurred about one month later, which is more or less the duration of *B. cucurbitae* cycle from egg to adult in Reunion Island (Vayssières 1999). In spite of the dry status of the corn plants, qualitative observations confirmed that adults could roost on them, finding food sources such as bird feces. In addition, our observations highlight the relevance of picking infested fruit fallen to the ground in order to reduce or to avoid the emergence of the following generation. Finally, our study suggests that local factors, such as availability of host fruit in the field, vegetative quality of roosting plants, and presence of food on the corn even if it is dry can affect the seasonal fluctuation of the populations of fruit flies.

Results clearly show the high variability of the communities of cucurbit flies in the different experimental designs where *B. cucurbitae*, *D. ciliatus,* and *D. demmerezi* coexist: (i) fluctuations of the populations of the different species were different from one site to another one and from one year to another one, (ii) relative abundances of the species changed according to the site and the year, and (iii) sex ratio varied from one experimental design to another and from one species to another. These results suggest that the characteristics of fruit fly communities are influenced by different factors including abiotic and biotic factors, acting globally or more locally.

One possible limitation of this study could be that it was restricted to austral summer and to a particular altitude range (from 750 to 1150 m). However, this season corresponds to the main period of cucurbit growing and to the period when the populations of fruit flies are at their highest (Vayssières and Carel 1999). Besides, this altitude corresponds to a major area of cucurbit crops (particularly zucchini and pumpkin) on Reunion Island. In addition,another limitation could be the low number (four) of experimental designs during the three—year study. Furthermore, although recording the number flies on corn plants by sight is a cumbersome and time consuming process, it is probably the more precise technique to study bioecological parameters such as density of populations or relative abundance, comparing different fruit fly species (with sex distinction) in different sites, years and dates. A third limitation could result from the fact that the design and the area (planted and observed) of the corn roosting sites differed (border, strip, patch) from one situation to another, and that two different cucurbit species (zucchini and pumpkin) were grown in our experiments.

Most studies on fruit fly communities have been done using traps and/or fruit collections, but these techniques have some limitations. For trapping, males of only some species are attracted by sexual parapheromone lures, while traps with food lures have limited attractiveness distance and are affected by competition with other nutrient sources. Besides, no correlation between the results of trapping and the real numbers of fruit flies has been demonstrated (Efrom 2009). For fruit collecting, a bias may come from the differences in the ability of the different fly species to develop in a particular host—fruit, and another bias may come from the difference between its abundance on corn and its low ability to develop in the cultivated fruit. In contrast, a few authors have chosen to use counting in their fruit fly studies: i.e., recently Yee (2002) on some species of *Rhagoletis* in USA, Brévault and Quilici (2007) on *Neoceratitis cyanescens* in La Réunion, Mausse and Bandeira (2007) on *Ceratitis capitata* in Mozambique, and Pinero et al. (2009) on *B. cucurbitae* in Hawaii. However, it is likely that the time required to count adults is adequately compensated by the large number of observations and high precision of records. In this study, time allocated to observation in the field required 147 hours (average of 35 minutes × 12 observations per day × 21 observations), and 18,441 fly adults were recorded in total. The methodology used in this study did not require the killing of any fly, and did not present any bias favoring one species or one sex. In addition, the real number of living adults (distinguishing species and sex on corn plants) was recorded. Finally, our study could take into account males and females of all the species (including *D. ciliatus* for which there is no parapheromone available) unlike trapping techniques. The comparison of these results with those obtained with other methodologies, such as adult trapping and emergence of pupae or adults from infested fruit collected in the fields, is of interest and importance. Indeed, we believe that results on real numbers of flies on roosting plants can provide complementary results and can cast new light on communities of fruit flies.

This study confirms that populations of adult cucurbit flies roost on corn plants placed within or around the crops. It suggests that corn can be considered as a trap plant for these Tephritids as already shown for *B. cucurbitae* in Hawaii (Nishida and Bess 1957; McQuate et al. 2003; McQuate and Vargas 2007). On Reunion Island, studies of attractiveness of different candidate trap plants for Cucurbit fly species have been recently performed, showing that corn is the most attractive plant for *B. cucurbitae* and *D. demmerezi* (Atiama-Nurbel 2012), unpublished data). This result leads us to associate a trap plant (corn) with spinosad— based bait treatment on this plant in an “assisted push-pull” system as already used for *B. cucurbitae* (Prokopy et al. 2003, 2004;Revis et al. 2004; Mangan et al. 2006; Vargas et al. 2008, 2009). This association represents a key technique proposed to manage Cucurbit fly communities on Reunion Island (Ryckewaert et al. 2010) and aligns with an agroecological approach in which sanitation, habitat management, and biological control are prioritized (Ferron and Deguine 2005; Deguine et al. 2009). Furthermore, this management technique using corn as a trap plant is particularly adapted in the context of Reunion Island, where adults of all the species roost on corn, although the characteristics of the communities vary.

This study provides new observations on the communities of Cucurbit flies on corn, but further research has to be implemented in order to obtain more knowledge on the bioecology of the three fruit fly species, such as daily rhythms and types of activities at different hours of the day. The results would help identify daily patterns of the three Cucurbit fruit flies, understand the distribution of adults observed in the agroecosystems, improve understanding of movements between border of corn and crop, and evaluate the localization and the level of damage in the field. In addition, future studies should be designed to confirm some of the hypotheses proposed in the present study: a single experiment comparing adults recorded *in situ* and adults emerged from fruit collected in the field; and dispersion (with marking—release— recapture techniques) of males and females of the different fly species in the presence or absence of resource plants (host plants, roosting sites).

Finally, our results on the distribution of adults will be compared with the results of ongoing studies using trapping (with sexual or food lures) or infested fruit collections. Moreover, we are confident that this methodology used on Reunion Island can be extended and adapted for use in future studies in the Indian Ocean or other parts of the world.

**Table 1.**
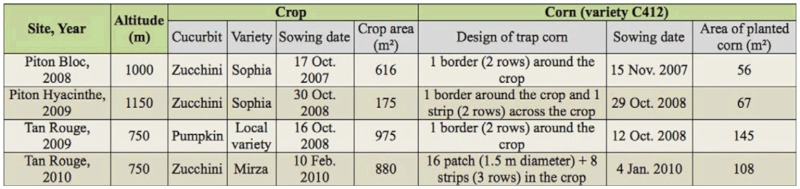
Cultural characteristics of cucurbit agroecosystems in which corn plants were planted.

**Table 2.**
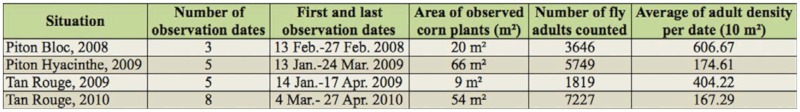
Period, frequency and area of observation, total numbers of adult flies recorded during the season, and average density (per 10 m^2^ of corn) of adults per date in each experimental design.

**Table 3.**
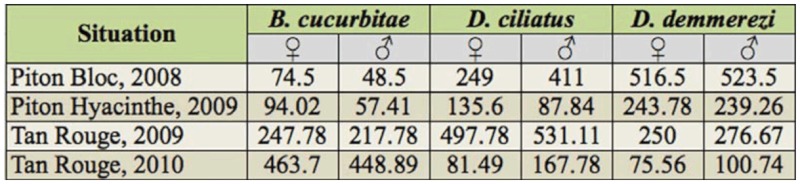
Density (per 10 m^2^ of corn) of adult flies, distinguishing species and sex recorded in the different experimental designs.

**Table 4.**
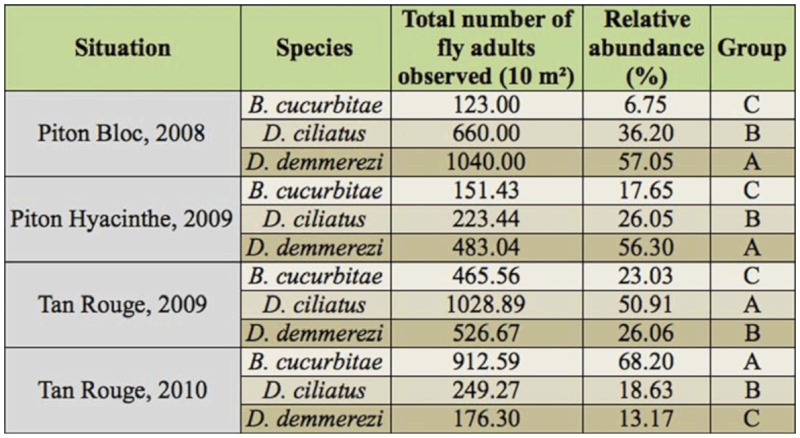
Total numbers of adult flies observed per 10 m^2^ and comparison of relative abundances of adults of *Bactrocera cucurbitae, Dacus ciliatus,* and *Dacus demmerezi* (per 10 m^2^ of corn) in each experimental design. Relative abundances not followed by the same letter in range are significantly different according to the test of pair—wise comparison of proportion with the Benjamin and Hochberg correction.
